# Failure Mechanisms and Changes in Load-Bearing Capacity of Sinusoidal Corrugated Girders Under Fatigue and Static Loading

**DOI:** 10.3390/ma18245614

**Published:** 2025-12-14

**Authors:** Krzysztof Śledziewski, Marcin Górecki

**Affiliations:** Faculty of Civil Engineering and Architecture, Lublin University of Technology, Nadbystrzycka 40, 20-618 Lublin, Poland

**Keywords:** sinusoidal corrugated-web girder, fatigue behaviour, crack initiation location, lateral-torsional buckling, hot-spot stress analysis, cycling load, changing load capacity

## Abstract

**Highlights:**

**What are the main findings?**

- Full-scale sinusoidal-web girders tested under static and fatigue loading.
- Two failure modes identified: local web buckling and lateral–torsional buckling.
- Fatigue life mainly governed by load range ΔF within the tested cycle range.

**What are the implications of the main findings?**

- Global stiffness remains stable until crack initiation, then degrades with damage.
- Nominal-stress design is conservative for the investigated welded flange details.
- Results support safer design of sinusoidal-web girders under repeated loading.

**Abstract:**

Steel girders with corrugated webs are increasingly used in bridge and building structures subjected to cyclic variable loads, where the geometry of the corrugation plays an important role in fatigue performance. This paper investigates the fatigue behaviour and failure mechanisms of full-scale steel girders with sinusoidal corrugated webs subjected to static and cyclic four-point bending. Five simply supported girders were tested: one reference beam under monotonic static loading, two beams under long-term cyclic loading with different load ranges Δ*F* and numbers of cycles *N*, and two beams subjected to cyclic loading followed by a static test to failure. The experimental programme focused on the influence of the load range Δ*F* and the number of cycles *N* on damage development, stiffness degradation and residual load-bearing capacity, as well as on the interaction between local web instability and global lateral–torsional buckling. The test results show that two main failure mechanisms may occur: (I) local buckling of the corrugated web combined with yielding of the flanges, and (II) a combined mechanism involving local web buckling and lateral–torsional buckling of the girder. For the investigated configurations and within the range of load ranges and numbers of cycles considered, the load range Δ*F* was found to be the dominant parameter governing fatigue damage, whereas the number of cycles had a secondary influence. The global stiffness of the girders in the elastic range remained almost unchanged until the late stages of loading, and even after pre-fatigue loading, the girders were able to carry a significant portion of their original ultimate load. The results provide experimental data and insight that are relevant for the fatigue assessment and design of steel girders with sinusoidal corrugated webs in bridge and building applications.

## 1. Introduction

Steel plate girders are the most commonly used elements in engineering structures. Considering weight optimisation, the central part of the cross section (web) is usually made as an element with a small thickness in relation to its height and length. This slenderness has a negative effect on its stability. Webs with high slenderness ratios are susceptible to buckling under the influence of shear, bending, or a combination of both. To eliminate the problem of local buckling of the web, its stiffness must be increased [1]. One solution is to increase its thickness. A more common approach is the use of transverse and longitudinal stiffeners, which provide the necessary shear strength to the beam in critical areas [2]. Ensuring both the stability of the web and shear capacity often leads to an uneven distribution of transverse ribs along the length of the beam. This not only disrupts the rhythm of the stiffening elements, which adversely affects the side appearance of the load-bearing structure, but also significantly increases the cost of manufacturing such girders.

To solve the problems associated with web buckling and the design, manufacturing, and welding of stiffeners, corrugated sheets are used instead of traditional flat webs [3]. Steel corrugated sheets with corrugated webs are very common in civil engineering. In addition to their use in general-purpose steel structures, they are employed in structures with significant dynamic properties. In recent years, steel girders with corrugated webs have been used as girders in bridge support structures and as crane girders in industrial facilities [4,5,6,7,8].

The main cause of damage to structures subjected to cyclic variable loads during everyday use is fatigue cracking. However, it should be remembered that the connection between the corrugated web and flanges changes from a simple to a curved welded connection, which significantly increases the degree of stress concentration owing to the change in geometry. Curved welding is also more difficult to perform, leading to more frequent welding defects. In addition, corrugated webs have a more complex stress state than that of classic flat webs.

As early as the 1970s, Harrison [9] conducted fatigue tests on two sinusoidal corrugated beams and concluded that their fatigue strength was 50–100% higher than that of traditional corrugated sheets. Korashy and Varga [10] conducted tests on four steel beams with different web stiffeners and found that beams with a corrugated web showed 25% higher fatigue strength than that of structures with a flat web. One of the earliest systematic experimental studies on the carrying capacity of girders with corrugated webs was presented by Pasternak and Branka [11], who investigated the ultimate behaviour of such members under static loading. Later, Dilger and Pasternak [12] reported static tests and a cyclic loading test (over 90,000 cycles) on girders with sinusoidal corrugated webs, providing important reference data for both ultimate and fatigue behaviour. Sause et al. [13] conducted experimental fatigue tests on eight full-size plate girders with trapezoidal corrugated webs made of HPS 485W steel. They showed that fatigue cracks were initiated in the web–flange welds, particularly in the areas of trapezoidal fold bends. Ibrahim et al. [14] observed the formation of a fatigue crack in the lower flange at the butt weld in the constant moment zone near the end of the inclined fold. Anami et al. and Anami and Sause [15,16] noted that the fatigue strength is negatively affected by the longitudinal fold of the corrugated web. Therefore, it is necessary to have a large bending radius so that the critical fatigue point on the inclined folds is far from the longitudinal folds. Kövesdi and Dunai [17] conducted experiments on six steel beams with trapezoidal corrugated webs and found that the loading method and weld size have a significant effect on the fatigue life. In addition, they showed that the web height and wave amplitude significantly influence the local stress concentration. Wei et al. [18] researched the evolution of fatigue cracks by analysing both experimental data and numerical simulations. They found that cracks are most often initiated in the crest of the web waves, especially near the welds. The fracture trajectory extends diagonally in the web–flange direction, which the authors attributed to the triaxial stress states. They also showed that local stress concentrations strongly depend on the wave curvature radius and type of the weld. Xu et al. [19] found that critical cracking occurs in the zone of constant moment, propagating through the shear force to the zone of interactive moment and shear. The stress concentration factor, related to the wave angle and bending radius, increases the fatigue life. Zhao et al. [20] conducted a comprehensive numerical analysis of fatigue crack propagation. The study focused on the centre-web weld details, where intense local stresses leading to cracking were observed. The results of the study showed a strong influence of the wave geometry and crack location on the crack propagation direction and fatigue life. Zhang et al. [21] performed a numerical simulation of the residual stress distribution characteristics of welding. They also derived the fatigue limit equations by considering the combined effect of residual welding stresses and vehicle loads. Liu et al. [22] investigated the fatigue properties of large-scale corrugated girders used in bridge construction. Based on the critical distance theory, they proposed a fatigue detail category corresponding to optimised corrugation angles. They showed that the point stress concentration factor at the web–flange weld edge increases with the corrugation angle and decreases with the bending radius. When the corrugation angle and bending radius are constant, the point stress concentration factor increases with the corrugation wavelength. Based on simplified T-shaped elements as substitute models for full girders with corrugated webs, Tong et al. [23] showed that the intersection of the horizontal and diagonal corrugations of the web is the most vulnerable fatigue zone. Zuo et al. [24] conducted tests on twenty corrugated beams with trapezoidal webs. The results of fatigue tests showed that among all tested samples exhibiting fatigue damage, 85% of the initial cracks occurred at the point S in the tension zone, whereas the remaining samples exhibited cracks at the edge of the web weld. The crack propagation process depends on the initiation point. Based on an analysis of 86 fatigue test results available in the literature, Hlal and Al-Emrani [25] proposed the fatigue categories for corrugated web girder details depending on the corrugation angle. It should be noted that the Eurocode 3 standard [26] does not contain a detailed category for the fatigue design of welded connections between the web and flange in beams with corrugated webs. Fatigue is also a critical issue for masonry structures. Recent studies have shown that ancient brick arch bridges and brick masonry elements subjected to repeated traffic loads may experience significant deterioration and that specific fatigue assessment methods are required for these materials [27,28]. Laterza et al. proposed a stress–life curves approach for the fatigue assessment of ancient multi-span brick arch bridges still in service [27], while Roberts et al. investigated the quasi-static and high-cycle fatigue strength of brick masonry specimens representative of old masonry arch bridges [28]. These works highlight that fatigue problems are relevant across different structural typologies, which further underlines the importance of improving the understanding of fatigue behaviour in steel girders with corrugated webs. Given the increasing use of corrugated web beams in bridges and other engineering structures, where these elements are subjected to long-term variable loads, and the variety of corrugation types used, it is necessary to expand the knowledge related to fatigue life.

The research presented in this article focused on determining the mechanisms of damage development in sinusoidal corrugated girders and assessing their behaviour under static and cyclic loads. The scope of the work included the analysis of the changes in the ultimate load-bearing capacity, stress and strain distribution, as well as the identification of typical forms of damage and their development depending on the amplitude and number of load cycles.

## 2. Materials and Methods

### 2.1. Experimental Programme

**Figure 1:**
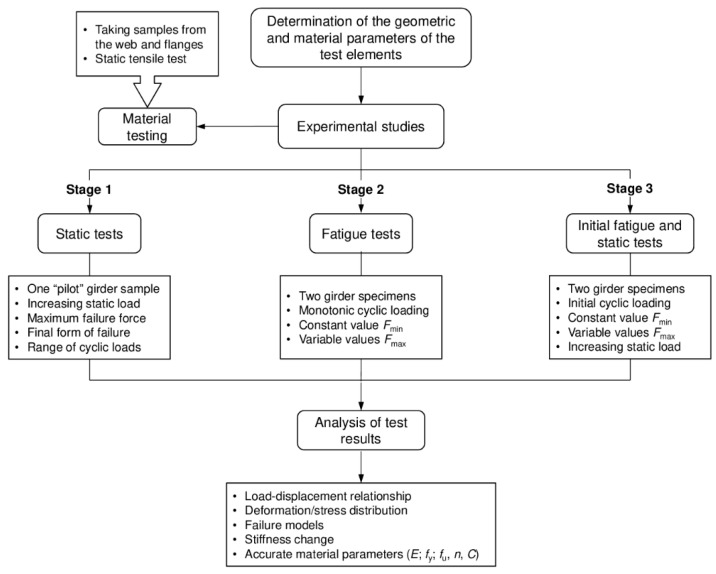
Block diagram of the experimental programme and methodology adopted in this study, showing the static reference test (girder sample 1), long-term cyclic tests with different load ranges Δ*F* and numbers of cycles *N* (girder samples 2 and 3), and preliminary cyclic loading followed by a static test to failure (girder samples 4 and 5).

In the context of ongoing research on the optimisation of steel elements used in load-bearing structures, this study was conducted in cooperation with the Kielce University of Technology and the Bridge Research Centre, a branch of the Road and Bridge Research Institute. The Kielce branch of the Road and Bridge Research Institute subjected five model girders with a scale similar to that of actual structures to experimental testing. The research programme consisted of three stages, as shown in Figure 1. All the elements tested had the same geometric and material parameters, as well as the same external load pattern.

In the first stage, specimen 1 was tested under monotonic four-point bending until failure. This static test served as a reference for the subsequent fatigue and post-fatigue tests and provided the ultimate load capacity *F*_g_ and the corresponding displacement and strain distributions.

In the second stage, specimens 2 and 3 were subjected to constant-amplitude cyclic (fatigue) loading to failure under four-point bending. For both girders the minimum load level was kept constant at *F*_min_ = 10 kN, while the maximum load, *F*_max_, and thus the load range, Δ*F* = *F*_max_ − *F*_min_, differed between the two specimens. The tests were continued until fatigue failure, providing two reference points on the *F*–*N* behaviour of the investigated welded details.

In the third and final stage, specimens 4 and 5 were first subjected to constant-amplitude cyclic loading not leading to failure, with the same minimum load *F*_min_ = 10 kN and the load range Δ*F* corresponding to that used in specimen 3. After a prescribed number of cycles *N*, the cyclic loading was stopped, and each girder was subsequently tested under monotonic four-point bending to failure. This makes it possible to assess the influence of prior fatigue loading on the residual stiffness and residual static load-bearing capacity of the girders.

Owing to changes in the strength parameters of steel as a result of cold forming of corrugated web sheets, after completing the experimental tests on the test specimens, additional tests were performed on steel from the sinusoidal webs and flanges to determine its exact mechanical properties.

### 2.2. Test Elements and Materials (Geometry and Fabrication)

As demonstrated by Abbas et al. [29,30] and our previous research [31,32], the scale of the model has a significant effect on the laboratory test results of elements with corrugated sheets. On this basis, it was determined that models similar to actual structures would be the optimal test elements. The tested girders are representative of medium-span steel members with sinusoidal corrugated webs typically used in bridge decks and industrial or transport-related buildings, where reduced self-weight, high shear stiffness and good fatigue performance under traffic loading are required.

**Figure 2:**
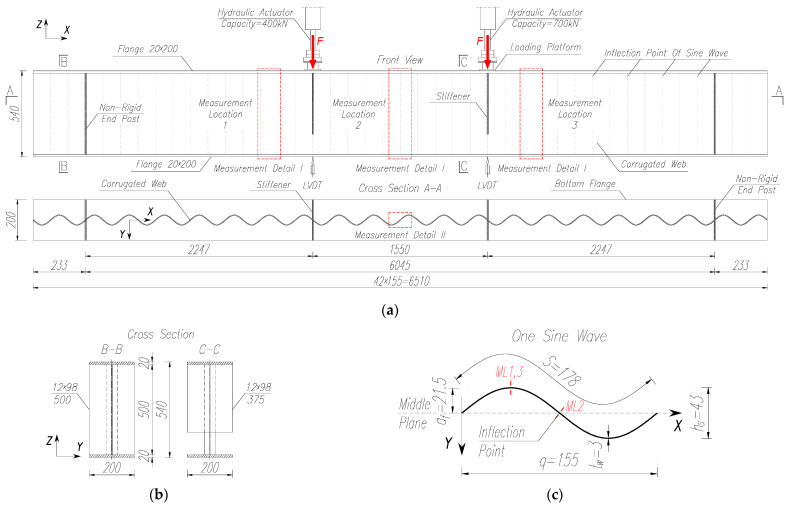
Geometry of the tested girder samples (units: mm): (**a**) front view and cross-section A-A; (**b**) cross section B-B and C-C; (**c**) view of a single sine wave.

The geometry and cross section of the test girders were designed in accordance with [26], aiming at a configuration representative of medium-span bridge girders. The span, web depth and sinusoidal corrugation parameters were selected within ranges commonly used in practice, while the flange dimensions were chosen so that the web remains slender and susceptible to local buckling under shear and combined bending–shear. At the same time, the overall member slenderness was set to allow for possible interaction between local web instability and lateral–torsional buckling under four-point bending. In this way, the specimens were intended to realistically capture the failure mechanisms and the influence of fatigue loading on girders relevant for bridge applications. The exact parameters of the tested girder samples are shown in Figure 2 and summarised in Table 1.

Ultimately, single-span I-beams with a total length of *L* = 6510 mm and span of *L*_0_ = 6045 mm were selected for testing. The girder samples were equipped with a web with a thickness of *t*_w_ = 3 mm and a height of *h*_w_ = 500 mm, made of cold-formed steel sheets. The width and thickness of the flanges were *b*_f_ = 200 mm and *t*_f_ = 20 mm, respectively. Their cross section was selected in such a way as to eliminate the risk of warping. At the points of support and external load application, the elements were reinforced with vertical single double-sided stiffeners made of 12 mm-thick flat sheets. Support stiffeners were provided along the entire height of the web. To minimise the effects of unfavourable fatigue details, the height of the stiffener under the load was limited to ¼ of the distance from the bottom chord (Figure 2b). Owing to the negligible values of the longitudinal circumferential stresses in the corrugated web panels [33], flexible support ribs were used [1,34]. The length of the plate end on each side was set to 233 mm. In all specimens, the transverse stiffeners located directly beneath the concentrated loads were intentionally discontinued a short distance below the top flange, i.e., they were not full-depth stiffeners; this configuration results in a less restrained web region under the point loads and may lead to a slightly conservative estimate of the ultimate load compared to girders with full-depth transverse stiffeners.

The test elements were manufactured on an automatic line at the steel structure manufacturing plant Zekon Sp. z o.o. in Ruda Śląska, Poland [35,36]. All test elements subjected to laboratory testing had the same corrugation profile of the web, which was obtained by deforming a flat sheet passed through specially adapted ring rollers. The geometry of the corrugated web profile is shown in Figure 2c. The wave amplitude was *a*_f_ = 21.5 mm, wave height was *h*_s_ = 43 mm, projection length of a single wave was *q* = 155 mm, and the wave length in the development was *S* = 178 mm.

The web with flanges was joined with a continuous single-sided fillet weld made using the metal inert gas welding method. The welding process was automated and performed by computer-controlled robots. By following the shape of a sinusoidal wave reflecting the movement of the sheet metal, the thickness of the weld was varied from 3.0 to 5.0 mm.

All test elements were made entirely of S355 steel. To determine the exact material parameters, additional steel strength tests were conducted on samples taken from beam fragments that were not subjected to excessive stress during the main tests. The discs of the beam webs for sample preparation were taken from sections where the curvature of the sine wave was closest to a straight line, i.e., from the so-called zero wave deflection section (Figure 2c).

The dimensions of the samples and the static tensile test procedure were in accordance with Eurocode 3 [37]. Table 2 summarises the average values of the measured mechanical properties of the steel used in the individual components of the beams, i.e., tensile strength (*f*_u_), yield strength (*f*_y_), and longitudinal elastic modulus (*E*).

The results obtained during the static tensile test also allowed the determination of the Hollomon parameters describing the relationship between strain and stress in the plastic range [38]. For this purpose, the tensile strengthening exponent (*n*) and strength coefficient (*C*) were determined in accordance with ISO 10275 [39]. The determined parameters are presented in Table 3.

### 2.3. Instrumentation and Measured Values

**Figure 3:**
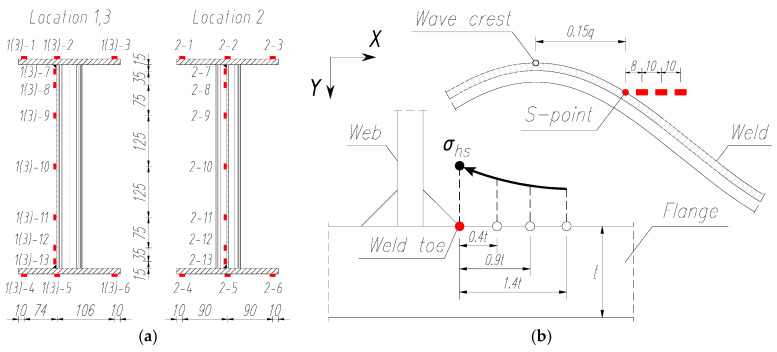
Position of strain gauges for measurements (units: mm): (**a**) normal deformations in the web and flanges, (**b**) geometric deformations in the tension flange.

During laboratory tests, surface deformations were measured in both the corrugated web and in the flanges. For this purpose, unidirectional foil strain gauges were used, which were placed on the test elements at three locations (Figure 2a). The first and third measurement sections were located in the bending moment and shear force zone (near the external loads) on the ‘ridge’ of the wave, where web damage was expected to occur. The second measurement section was located in the middle of the beam span in the zero wave deflection section (Figure 2c) marked by a constant bending moment. The exact location of the strain gauges is shown in Figure 3a.

At the height of the web, seven measurement points were set in each cross section. All strain gauges on the web were placed on the side of the connection with the flanges of the box girder using welds. Three strain gauges were used on each flange in each measurement cross section, giving a total of 39 measurement points. It should be noted that not all five girder samples were analysed with the same strain gauge arrangement owing to the nature of the tests performed in each stage. Only the pilot sample 1 was tested using the above method.

In the case of test specimens 2 and 3, fatigue cracks in the pure bending zone could possibly lead to failure. Therefore, in accordance with the study by Zuo et al. [40], the position of point S was determined in this area, at the point of stress concentration, to determine the geometric stresses in the so-called hot spots (Figure 3b). The measurement was performed by following the recommendations of Hobbacher and Baumgartner [41] by using three unidirectional strain gauges, arranged in the longitudinal direction, at reference points 0.4*t*, 0.9*t*, and 1.4*t* (where *t* is the thickness of the flange). Point S on the tensioned belt in the corrugated sheet metal beams is the most critical point of stress concentration, where fatigue cracks are the most common [42].

In the stage 3 test specimens (Nos. 4 and 5), the number and placement of the strain gauges was reduced to two measurement sections. One was located in the middle of the beam span and the other in the area of external load transfer (Figure 2a and Figure 3a).

In addition, during the tests, the vertical displacements of the test elements were recorded synchronously with the applied load using inductive sensors (LVDT). The measurement points were located on the underside of the lower belt located under the points of application of the forces, as shown in Figure 2a.

### 2.4. Test Configuration and Loading Procedure

**Figure 4:**
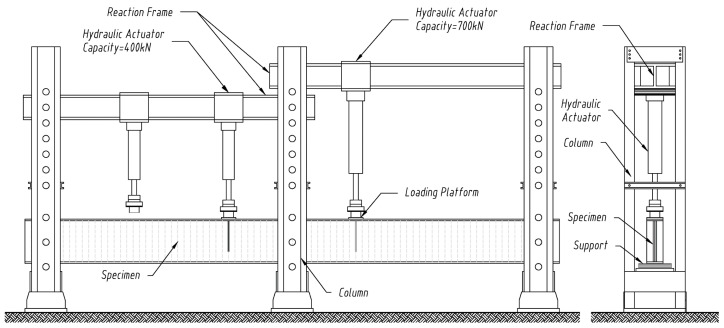
Schematic of the four-point bending test rig for the corrugated-web girders.

The test elements were placed between the posts of a steel frame, which, in the event of the beam slipping off one of the bearings, prevented it from moving from its central position (Figure 4). The girder samples were supported on one side by a non-sliding articulated support and on the other side by a sliding articulated support.

The points of application of the external loads were selected in such a way that the value of the bending moment generated at the centre was constant, that is, a four-point scheme. The load in the form of two concentrated forces was applied at a distance of 2480 mm from the ends of the test model, and the distance between them was 1550 mm (Figure 2a).

The external load was applied using two hydraulic actuators with a force of 400/700 kN. Additionally, the forces were applied to the upper flange of the plate beam via a steel plate, which distributed them to a band transverse to the beam axis with dimensions of 140 × 200 mm, as shown schematically in Figure 4.

The process of loading the girders depended on the methodology adopted and the stage of testing (Figure 1). Before proceeding to the main tests in each stage, each sample was first preliminarily loaded statically to determine the static response of the girder by measuring the deformations and displacements at various measurement points (Figure 2a and Figure 3a).

The static load was increased through ‘loading–unloading’ cycles. In each subsequent cycle, the force increased by 25 kN until the sample was completely destroyed. After reaching the minimum or maximum load value in a given cycle, the force was maintained at a constant level for approximately 180 s. During this time, the readings from the measurement devices were recorded and the changes occurring in the test elements during the test were observed. The procedure for increasing the static load was the same as that used in the tests in stages 1 and 3, i.e., for sample 1 (pilot) and samples 4 and 5 (pre-loaded cyclically).

To avoid resonance while maintaining the loading efficiency in all fatigue tests, a test frequency of 2 Hz was applied. In stage 2, it was assumed that the fatigue tests would continue until there was a significant decrease in load or cracks appeared in samples 2 and 3. For samples 4 and 5 (stage 3), fatigue testing was conducted until ½ of the number of cycles meeting the failure criterion for identical girder samples in stage 2 was reached. The deformation and deflection of the samples were measured at regular cycle intervals.

The fatigue load ranges Δ*F* used in the tests were selected so that the resulting stress ranges in the critical welded details (web–flange and web–stiffener joints) are representative of the upper part of the spectrum that may occur in real bridge girders and crane girders under heavy traffic or lifting operations. At the same time, the load levels were chosen to remain compatible with the capacity of the test rig and with the objective of completing the tests within a reasonable time. The experimental programme was therefore not intended to reproduce a specific traffic or crane load history, but rather to investigate the behaviour of sinusoidal corrugated-web girders under constant-amplitude cyclic loading with realistic stress ranges.

## 3. Results and Discussion

### 3.1. Static Load Results

#### 3.1.1. Displacement Versus Load Relationship

**Figure 5:**
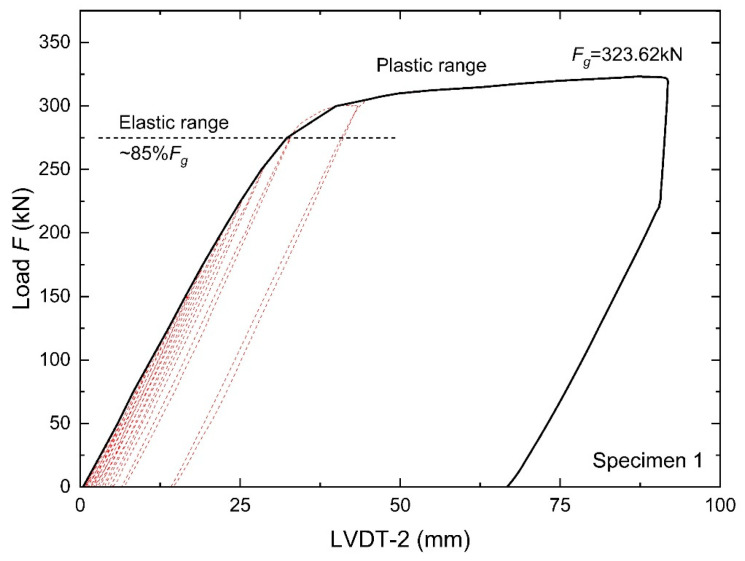
Force–displacement curve of girder sample 1.

The stage 1 test programme involved testing a pilot sample in a four-point bending test by applying increasing static load in successive cycles until the element failed. As shown in Figure 2, vertical displacements were measured under each external force, in the axis of the lower flange. During the test, the highest displacement value was observed at the LVDT-2 measurement point. The obtained load–vertical displacement curves are presented in Figure 5.

Starting from zero *F*, a slight but steady increase in the permanent deflection is observed in the successive loading–unloading cycles. A permanent displacement of 6.87 mm of the sheet metal in its plane is observed only after exceeding the elastic range (the zone showing linearity of the curve) of approximately 85% of the limit force (*F*_g_). At this load level, the stability of the corrugated web begins to reduce, which is indicated by changes in the geometric shape of the wave. From this point on, the curve becomes nonlinear, with a clear increase in the displacement under a slight increase in load until the ultimate load capacity (*F*_g_) is reached. After exceeding the ultimate load capacity, the force value drops rapidly and the deflection of the beam decreases. The ultimate load value at specimen failure was recorded as *F*_g_ = 323.62 kN.

#### 3.1.2. Mode and Propagation of Failure

**Figure 6:**
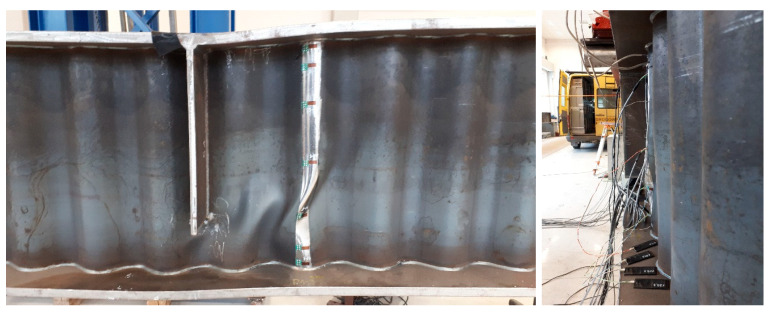
Final form of failure of girder sample 1.

The destruction of the test element occurred as a result of local instability of the web in the area subjected to the bending moment and constant transverse force (Figure 6). The intermediate stiffener, located in the axis of force application and in the immediate area of destruction, remained intact.

**Figure 7:**
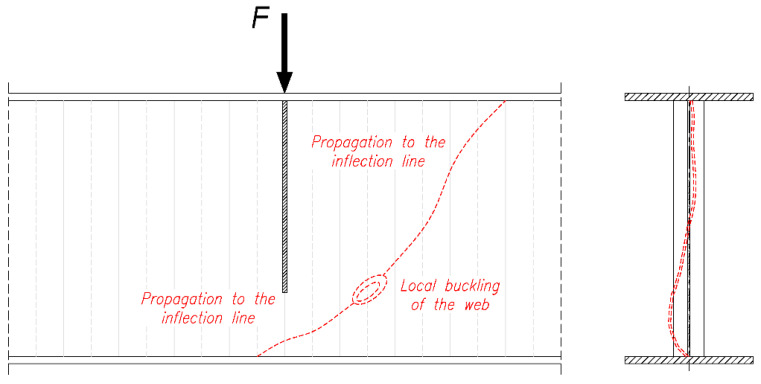
Local loss of stability of a sinusoidal corrugated web subjected to shear in the region under the concentrated load, showing the development of local buckling in the web and the influence of the shortened transverse stiffener located beneath the load.

The process of loss of stability of the corrugated web of the stage 1 girder sample is shown in Figure 7. In the initial phase, it begins with the formation of local sinusoidal buckling of the panel in the straight section between the adjacent corrugations. The first local point of loss of stability occurs between the tension chord and neutral axis of the girder. After the formation of a local buckling centre, a plastic buckling line is formed, followed by immediate loading of the beam flanges with a transverse force. This causes the flanges to buckle in the plane of the girder. It should be noted that the initial phase of local instability was mild, with sudden plastic buckling occurring in the final phase. As shown in Figure 7, the buckled zone propagates beyond the position of the shortened transverse stiffener located under the concentrated load, which reflects the reduced restraint provided by this non-full-depth stiffener detail.

#### 3.1.3. Deformation Distribution

**Figure 8:**
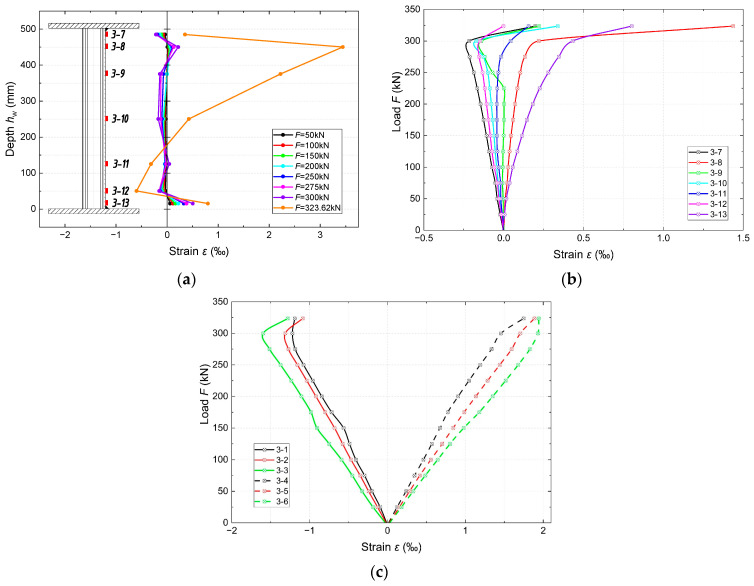
Measured longitudinal strains ε at location 3 of girder sample 1 under monotonic four-point bending: (**a**) distribution of longitudinal strain over the web height at selected load levels; (**b**) load–strain response in the web (strain gauges 3-7÷3-13); (**c**) load–strain response in the flanges (strain gauges 3-1÷3-6, solid lines—compression flange, dashed lines—tension flange). Positive strains denote tension.

Figure 8 show the deformations measured during the test. The location of the measurement sections and markings of the individual measurement points are consistent with those in Figure 2 and Figure 3.

It can be observed that in the initial stage of loading, in the web located in the zone of bending moment and shear force (Figure 8a), a slight but steady increase in the deformation (*ε*) occurs until the onset of instability of the corrugated web (~85% of the destructive force, *F* = 275 kN). Further into the loading phase, a strong nonlinearity and height gradient appear. At the upper measurement points, the *ε* value increases significantly, and the curve becomes asymmetrical. This indicates the development of diagonal stress fields on the force-support line. At a load equal to the ultimate load capacity (*F*_g_), the deformation curve corresponds to the observed bending of the fold and buckling of the web (Figure 6). The *ε* values at the individual measurement points on the web are shown in Figure 8b. In the elastic range, the relationships are mostly linear, with varying sensitivity. The rate of increase in deformation at the individual measurement points depends on their proximity to the shear paths. Until the onset of web instability, the chords behave quasi-linearly, which confirms that the normal stresses from the bending are mainly absorbed by the chords (Figure 8c). After exceeding the value of 0.85*F*_g_, a clear deviation from linearity and a shift in the neutral axis towards the compressed chord are visible. The maximum deformation values reach 2.0‰ in the tension chord and 1.6‰ in the compression chord. The chords enter the plastic range in the final stage of loading (destruction).

Deformation measurements (*ε*) in the middle of the span confirm that the bending moment is transferred only by the flanges, and the stresses in the corrugated web are negligible in the pure *M* zone. The deformations at the measurement points located in the immediate vicinity of the chords show higher values (*ε)*, with only the point near the upper chord showing significantly greater compressive deformations with increasing *F*. At the other points, the deformations oscillate around zero throughout the load range. In the case of the belts, a linear relationship can be observed up to a value of 275 kN, with symmetrical tension of the lower belt and compression of the upper belt. In the later stages of loading, a deviation from linearity and the onset of plasticity of the belts (values of ~2‰) can be observed. The differences in the *ε* values between the individual points across the width are insignificant. This deformation pattern is consistent with the typical mechanical characteristics of a bent element.

### 3.2. Monotonic Load Results

#### 3.2.1. Displacement Versus Load Relationship

Fatigue tests were conducted for various load ranges. Table 4 summarises the cyclic load parameters for both stage 2 and stage 3 tests. *F*_max_ (*F*_min_) denotes the maximum (minimum) cyclic load value, and Δ*F* denotes the load range.

The minimum load level for all elements was 10 kN. The maximum load value depended on the destructive force obtained in the stage 1 tests. Ultimately, it was assumed that *F*_max_ would be 0.8 (260 kN) and 0.4 (130 kN) of the destructive force for girder samples 2 and 3 and 4 and 5, respectively. However, during the fatigue tests, sample 3 reached the set number of limit cycles (2 × 10^6^) for structural elements [26]. The maximum load was then increased to 0.6 (195 kN) of the destructive force. In the case of beam 5, a load range of 10–195 kN (0.6*F*_g_) was already used in the preliminary fatigue tests.

**Figure 9:**
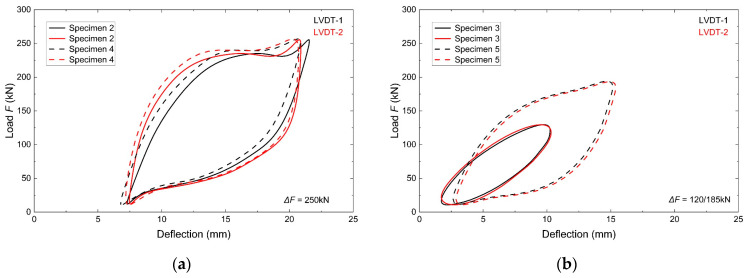
Cyclic deflection curves: (**a**) samples 2 and 4, (**b**) samples 3 and 5.

The measured deflections of the measurement points of the test elements during monotonic loading and unloading tests are presented in Figure 9. The samples were subjected to loads at different levels, depending on the stage of testing, which is reflected in the resulting hysteresis loops. Samples 2 and 3 were tested at a high load range (Δ*F* = 250 kN). In contrast, samples 4 and 5 were tested at lower load ranges of Δ*F* = 120 and 185 kN, respectively.

It can be observed that the samples of girders 2 and 4 are characterised by a wide loop, with a strong gap between the loading and unloading paths (Figure 9a). The strong nonlinearity of the curve and high value of permanent deflection (7.59 mm) clearly indicate local elastic-plastic behaviour of the beam during the tests (in the area of external load application). Sample 3, which was initially subjected to a load of Δ*F* = 120 kN, worked mainly elastically (Figure 9b). The permanent deflection was small (2.06 mm). The shape of the loop is almost oval (elliptical) and narrow, with little hysteresis. However, with an increase in the load range to Δ*F* = 185 kN, the permanent deflection increases to 3.46 mm because of significant plastic deformation. An increase in hysteresis is also visible. The loop is significantly wider with a clear gap between loading and unloading. As in the case of elements 2 and 4, the samples of girders 3 (after load change) and 5 exhibit mixed behaviour during the tests, with a large local plastic contribution.

The subsequent analysis of the obtained loops reveals that the deflection values measured at the load points differ from each other. This can be attributed to the influence of the separate operation of the pistons of each actuator. Nevertheless, these differences were small, ranging from 0.50 mm to 1.27 mm. This phenomenon was constant and occurred in all the tested elements.

#### 3.2.2. Failure Modes and Fatigue Crack Propagation

Stage 2 fatigue testing was conducted until the load decreased significantly or clear cracks appeared in the girder samples. Fatigue cracks occurred in the web in both girder samples in the external load zone at the connection point with the stiffener. Sample 2 failed because of fatigue cracking at 454,636 cycles, whereas sample 3 showed cracks when the number of cycles reached 2,462,179. It should be noted that the number of load cycles for sample 3 exceeded the standard requirement of two million, which was because of the significantly lower load range than that of sample 2.

It can be seen that a fatigue crack is formed at the edge of the weld between the web and stiffener, on the bending-stress side. This is the location of the initial defect or stress-concentration zone. The crack is semi-elliptical in shape from start to finish and begins at an angle of approximately 45° to the weld line. As the crack propagates, the web at this location is completely torn apart. The upper surface of the crack is relatively smooth and flat, corresponding to the crack propagation zone resulting from the continuous opening and closing of the crack and mutual friction after a long period of cyclic loading. The lower part of the surface is rough because of the final fracture after excessive crack propagation.

The mechanisms of initiation and propagation of fatigue cracks in girder samples 2 and 3 can be summarised as follows: the fracture starts at the edge of the weld connecting the stiffener to the web, propagates towards the centre of the specimen under cyclic loading, and finally leads to a brittle fracture zone after excessive crack growth.

#### 3.2.3. Stress Distribution

The stress distributions in the flanges and web at regular intervals/cycles were assessed using the sensor readings. The stress values were determined based on the measured deformations using Hooke’s classical law, considering the elastic state of the tested girder samples, and based on the Ramberg–Osgood model considering the plastic state [43]. The Ramberg–Osgood equation is expressed using Hollomon’s parameters as

(1) ε=σE+σC1n,

where *ε* is the total deformation, *σ* is the stress, *E* is Young’s modulus, and *C* and *n* are the strength coefficient and hardening coefficient of steel, respectively.

**Figure 10:**
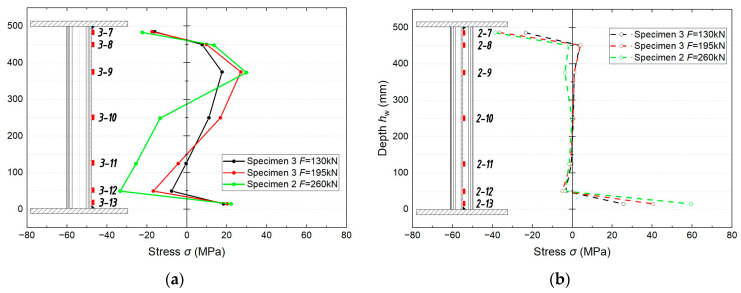
Distribution of normal stresses along the axis of the corrugated web of the tested specimens in the zone of (**a**) bending moment and transverse force, (**b**) constant bending moment.

Figure 10 show the stress distribution in the examined samples of girder Nos. 2 and 3 depending on the load value *F* and location of the measurement cross section. The distribution of normal stresses along the axis of the corrugated web in the zone of constant bending moment and transverse force (location 3, Figure 2) obtained during the tests are shown in Figure 10a. The distribution is clearly nonlinear, especially in the middle part of the height. The stress values are relatively low, indicating completely elastic behaviour. The stress distribution pattern reflects the influence of the web corrugation, which causes local compression and tension zones instead of a simple linear distribution as in traditional flat webs. This confirms that the web operates mainly under the shear forces. In sample 3, as the load increases, the stresses do not increase proportionally and locally ‘move’ up and down the web, indicating a redistribution of the forces. The stresses at a load of *F* = 130 kN are still relatively low and have a moderate gradient. However, changing the load to 195 kN increases the stresses on the tensile side, especially in the upper part of the web. The stress distribution in sample 2 at *F* = 260 kN is very nonlinear. Higher compressive stresses appear in the lower part whereas clear tensile stresses appear in the upper part.

Figure 10b shows the distribution of normal stresses along the axis of the corrugated web in the cross section located at the middle of the beam span (location 2, Figure 2) in the zone of constant bending moment. The stress values are very small, close to zero in the vicinity of the neutral axis, and practically unchanged at the height of the web. The curve resembles vertical lines. The distributions for sample 3 at different values of *F* are almost parallel and proportional to the load value. The wavy shape eliminates the longitudinal load-bearing capacity of the web, making the stress values negligible, and instead of a clear distribution, we see only minor disturbances in the form of local ‘bulges’. This confirms that the flanges carry almost the entire bending moment (*M*).

Measurements of the distribution of normal stresses in the flanges of the tested girders, depending on the position of the analysed cross section, show similar qualitative trends to those observed in the web. In the middle of the span of the analysed systems, the distributions are almost perfectly linear and symmetrical, and the differences in stress across the width are very small. Irregularities appear in the zone of constant bending moment and transverse force, mainly in the upper flange, where the local effects of the corrugated web disturb the stress distribution.

In cross section 1 in the region of combined bending moment and shear, in the upper (compressed) zone, the stress values are higher at the contact with the web and decrease towards the edge (differences of 10–20 MPa, 5–8%). A similar trend can be observed in the lower zone. The tension is greater at the web than at the edge (15–20 MPa, 6–7%). The upper flange is more sensitive; hence, it shows slightly greater unevenness than that of the lower flange. However, it should be noted that the stress distribution is consistent between the flange. In both samples at the given *F* levels, the stresses are of the same order. However, the values in the upper zone are higher. In the case of sample 3, the differences increase proportionally with the load. It should be noted that this is typical for a cross section in a zone with *V* ≠ 0. The maximum stress values are significantly lower than f_y_.

In location 2 in the pure bending moment zone, the stress distributions are consistent with each other and are symmetrical with very little heterogeneity across the width, of the order of 3–5% in the lower flange and 3–4% in the upper flange. In location 1, the maximum stress values are clearly lower than the yield strength (f_y_). In the case of sample 3, in both flanges, the distributions are parallel and proportional to the load *F*. The bending symmetry is maintained.

Additionally, in the zone of constant bending moment, the deformation values were measured at three consecutive positions in a specific area (extrapolation) at the edge of the weld connecting the lower flange with the web (Figure 2a and Figure 5b). Then, by extrapolation and interpolation, the structural stresses at the hot spot (*σ*_hs_) at this location were calculated using Equation (2):

(2) $$\sigma_{\mathrm{hs}}=2.52\sigma_{0.4t}-2.24\sigma_{0.9t}+0.72\sigma_{1.4t}.$$

The obtained structural stress values, together with the normal stress determined in accordance with Eurocode 3 [1], show that the nominal longitudinal stress *σ*_x_ on the upper surface of the lower flange in the middle of the span (calculated as *σ* = *My/I*, without considering the web) is higher than the measured hot-spot stress *σ*_hs_ for the load levels *F* = 130, 195 and 260 kN. This indicates that, for the investigated details, the nominal-stress-based design approach is conservative but safe with respect to bending strength. The most favourable stress distribution in the weld zone was obtained for the lowest load level (sample 3 at *F* = 130 kN), whereas higher load levels led to an increase of *σ*_hs_ relative to *σ*_x_ and to more pronounced local stress concentration in the weld zone. It should be noted, however, that as indicated in several studies [44,45], the relationship between nominal and design stresses is not always clearly defined and depends on the local geometry of the detail and on how the nominal cross section is defined. A local stress concentration appears in the weld zone.

#### 3.2.4. Evolution of Stiffness

During the cyclic loading and unloading process, the formation and propagation of fatigue cracks may change the stiffness of the system under consideration. The stiffness *K*_i_ of the girder samples in the i-th load cycle can be approximately taken as the ratio of the applied load range Δ*F* to the deflection range Δ*δ*, as follows:

(3) $$K_{i}=\frac{\Delta F_{i}}{\Delta \delta_{i}}=\frac{F_{i,\max}-F_{i,\min}}{\delta_{i,\max}-\delta_{i,\min}},$$

where *F*_i,max_ and *F*_i,min_ are the maximum and minimum values of the applied load, and *δ*_i,max_ and *δ*_i,min_ are the maximum and minimum values of the deflection, determined as the average of the measurements from two sensors located under the points of application of the forces in the i-th load cycle.

**Figure 11:**
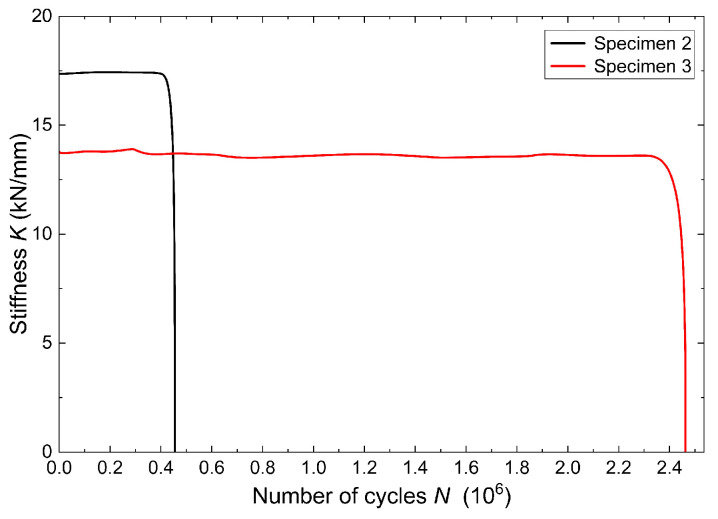
History of stiffness of the tested girder samples.

The change in stiffness of the tested girder samples with advancing load cycles is shown in Figure 11. It should be noted that the stiffness remains relatively constant before the occurrence of fatigue failure, although there are minor changes resulting from micro-damage, which are not visible macroscopically. Sample 2 shows a sudden loss of stiffness after reaching 454,636 cycles, which is attributed to the high load amplitude. In contrast, sample 3 shows a mild loss of stiffness after reaching 2,462,179 cycles, which can be attributed to the slow rate of crack initiation and growth because of the lower load amplitude.

### 3.3. Monotonic and Static Load Results

#### 3.3.1. Displacement Versus Load Relationship

The stage 3 girder samples were subjected to preliminary simulated cyclic loading, followed by increasing static loading until complete failure. The number of loading–unloading cycles (*N*) was selected on the basis of fatigue tests on identical samples from stage 2. Hence, *N* was set to 223,200 at Δ*F* = 250 kN and 1,250,000 at Δ*F* = 185 kN for samples 4 and 5, respectively.

**Figure 12:**
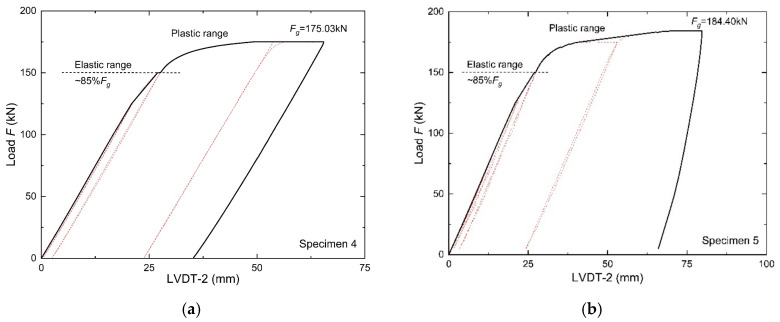
Force–displacement curve: (**a**) sample 4, (**b**) sample 5.

The load–vertical displacement curves of the girder samples obtained during the static tests at the LVDT-2 measurement point are shown in Figure 12.

It can be observed that the curves of both samples are convergent and generally correspond to the curve for the reference sample shown in Figure 5. The characteristic initial linear force–deflection relationship, as in the case of sample 1, occurs up to a level of ~85%*F*_g_. In this range, a slight increase in the permanent deflection is visible. Permanent displacements of 2.54 mm (sample 4) and 3.37 mm (sample 5) are observed only after the elastic range is exceeded. From this point onwards, the curve becomes nonlinear, but for sample 4, the plastic range is shorter and less pronounced than that in sample 1 (Figure 12a). The reserve load-bearing capacity after exceeding the elastic limit is small. In addition, a large increase in displacement is observed with a slight increase in load (*F*). After reaching the limit load (*F*_g_), a rapid loss of load-bearing capacity occurs at moderate deflections. Sample 5 demonstrates a more developed plastic range than that of sample 4 (Figure 12b). A greater increase in the displacement of the plate in its plane is observed, with a simultaneous greater increase in the load value (*F*) until the ultimate load capacity (*F*_g_) is reached. The loss of load-bearing capacity occurs more slowly than in sample 4, but with greater deflections.

Despite the overall similarity of the static equilibrium paths of samples 4 and 5, there are visible differences in the limit values. Table 5 summarises the limit values of the specimens, including the ultimate load capacity *F*_g_, onset of nonlinearity at 85%*F*_g_, and maximum deflections at *F*_g_ and 85%*F*_g_. The limit values obtained for sample 1 are also listed for reference.

It should be noted that the ultimate load capacity (*F*_g_) after fatigue is determined to a greater extent by the range of cyclic loads (Δ*F*) than by the number of load cycles (*N*). In the case of sample 4, a 46% decrease in the load-bearing capacity was observed when compared with that of sample 1. Despite a much higher number of cycles (*N*), sample 5 had a 5% higher load-bearing capacity than that of sample 4 and a lower decrease in *F*_g_ (43%) when compared with that of the reference sample. The same trend can be seen in the case of the limit deflection (*δ*_max_). During the tests, sample 5 achieved a significantly higher *δ*_max_ value (79.76 mm) and therefore a slightly longer plastic range than that of sample 4 (65.47 mm). However, these values were much lower than that of the reference girder sample, where the ultimate deflection was 91.82 mm.

Both girder samples also achieved a significantly lower threshold for transition to the nonlinear range (a clearly lower force of 0.85*F*_g_ at smaller deflections *δ*_0.85Fg_). This indicates a reduction in the effective stiffness of the systems under consideration owing to the initial cyclic loading and a faster onset of damage, leading to the complete destruction of the girder samples.

#### 3.3.2. Mode and Propagation of Failure

The destruction of girder sample 4 occurred in a manner similar to that of the reference element (Figure 6 and Figure 7) because of the local instability of the web in the region of transverse force loading. The intermediate stiffener, located under the force and in the immediate area of destruction, also remained intact.

The final form of damage is characterised by local buckling of the panel on the straight section between adjacent waves, which is shifted upwards to a greater extent than in sample 1. This was caused by faster plasticisation of the upper flange, leading to a shift in the axis of inertia towards the compression flange. As the web instability developed, the buckling spread to the entire height of the panel and to the adjacent folds. There were no signs of global buckling or loss of web stability. As in sample 1, the plastic buckling line runs diagonally from the lower flange (in the compression zone) to the upper flange. No clear fatigue cracks were found. The effect of the initial cyclic loading was mainly to accelerate the initiation of local web instability.

**Figure 13:**
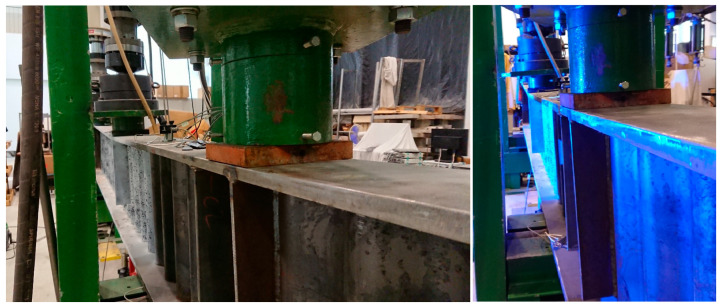
Final form of destruction of girder sample 5.

Sample 5 suffered global failure as a result of the loss of torsional and bending stability of the entire system [46,47,48,49,50]. The final form of failure obtained during the tests is shown in Figure 13. The rotation of the flanges relative to each other, twisting of the web, and displacement of the load nodes out of the beam plane are clearly visible.

The process of failure of sample 5 was completely different from those of samples 1 and 4. With increasing load (bending moment), the lateral displacements of the compressed flange (upper) begin to increase, initially by a very small extent, amplified by geometric imperfections. Failure is initiated when the upper flange, instead of continuing to work axially, tilts sideways and begins to rise diagonally. This is accompanied by the twisting of the entire cross section around the longitudinal axis. The lower flange moves in the opposite direction, and the web is twisted and deformed. After the initiation of torsion, the test element does not immediately lose its load-bearing capacity. Instead, a post-critical state develops. Bending in the horizontal plane occurs in the upper flange, whereas tension with a torsional component occurs in the lower flange. The corrugated web does not inhibit rotation but rather facilitates torsional deformation. As a result, with greater deflections, a combination of rotation and corrugation of the web is observed. As the torsion increases, the edges of the flanges, especially the compressed upper flange, become plasticised. Local bulging of the upper flange appears, and the cooperation between the flange and web disappears. This local bulging results from the concentration of compressive stresses near the web–flange junction and the loss of support provided by the corrugated web after local buckling, so that the slender flange plate undergoes out-of-plane plate buckling. Residual welding stresses and the flange plate slenderness further promote this deformation pattern. Ultimately, the girder sample is unable to withstand further increase in the load and is completely destroyed because of lateral torsion of the cross section around the longitudinal axis.

Preliminary long-term cyclic loading at a lower fatigue load level (Δ*F*) than that in girder sample 4 did not cause dominant local deformations of the web. However, it accumulated residual torsional deformations and, consequently, slightly weakened the torsional stability overall. In the final stage of loading, pronounced local bulging of the compression flange near the web–flange junction was observed, resulting from the concentration of compressive stresses in this region and the loss of support provided by the corrugated web after local buckling. It is worth noting that in comparison with a flat web, a corrugated web reduces the torsional stiffness of the cross section, which further increases the susceptibility of the beam to loss of torsional bending (LTB).

In the present experimental programme, the girders were laterally restrained at the supports and at the points of load application, and torsional rotations were not directly measured. Nevertheless, qualitative observations indicate that torsional effects had a limited influence on the bending capacity in most tests, where failure was governed by local web instability. In contrast, in specimen 5 the ultimate behaviour involved a clear interaction between local web deformation and global lateral–torsional buckling of the girder, leading to failure at an ultimate load equal to about 57% of the reference static capacity but with a markedly different deformation pattern. This highlights that torsional effects can become dominant when the load history and residual deformations reduce the effective lateral and torsional stiffness of the system.

#### 3.3.3. Deformation Distribution

Because of the initial high-amplitude cyclic loading, the upper flange of girder sample 4 underwent early plasticisation and faster local weakening of the web. Long-term cyclic loading with a lower cycle amplitude, as applied to sample 5, caused asymmetrical stretching of the web and, consequently, susceptibility to global torsion.

The deformation values measured during the tests in two measurement sections of girder sample 4 indicate that residual fatigue deformations appear in the form of small initial deviations. At lower load levels than in the reference sample, the deformations at the upper measurement points of the web transition to compression more quickly and exhibit earlier nonlinearity. After exceeding 0.85*F*_g_, the upper flange shows the onset of plasticisation, while the lower flange remains predominantly linear and the neutral axis of the cross section shifts upwards. In both the compressed and tensile zones, a clear uneven distribution of the deformation is visible. This behaviour is fully consistent with the final form of destruction and the mechanisms of its development described for girder sample 4.

**Figure 14:**
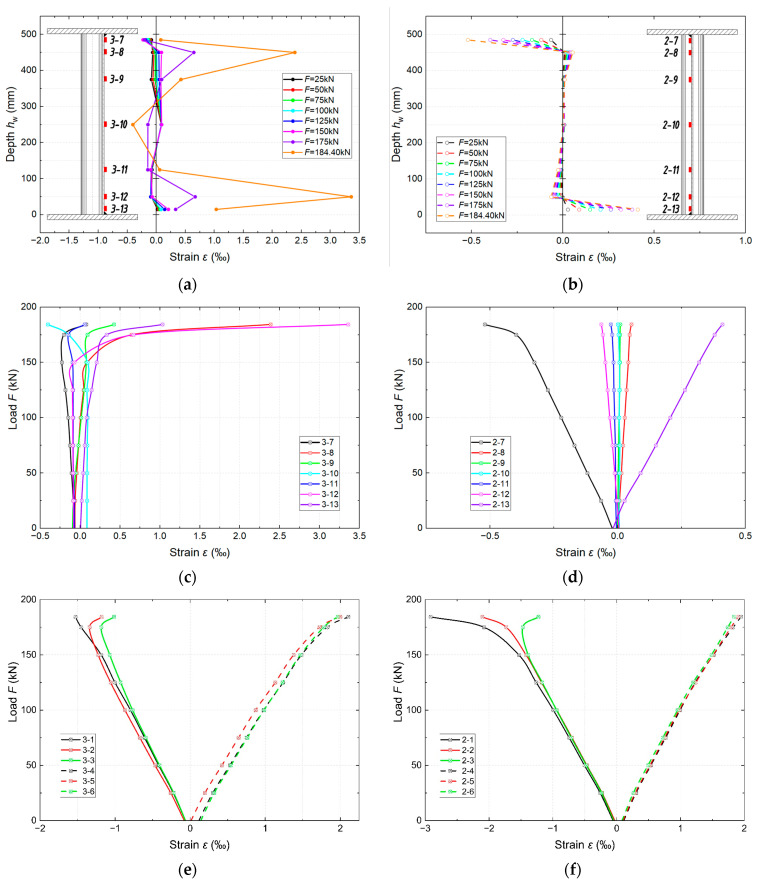
Measured longitudinal strains *ε* in girder sample 5 under the applied loading: (**a**) distribution of longitudinal strain over the web height in the region of combined bending moment and shear; (**b**) distribution of longitudinal strain over the web height in the region of constant bending moment; (**c**) load–strain response in the web in the region of combined bending moment and shear; (**d**) load–strain response in the web in the region of constant bending moment; (**e**) load–strain response in the flanges in the region of combined bending moment and shear; (**f**) load–strain response in the flanges in the region of constant bending moment. Positive strains denote tension.

Figure 14 shows the measured deformations in girder sample 5. In measurement location 3, the distribution of the deformation along the web height at lower load phases is typical for a corrugated web and is characterised by small *ε* values with variable signs (Figure 14a). After exceeding *F* = 0.85*F*_g_, a strong divergence is visible. Especially at the measurement points located below the neutral axis, the deformations change to large positive values, whereas they remain slightly negative at the points located in the upper part of the web. The web is stretched on one side and compressed on the other.

A rapid increase in the web deformation at the bottom chord and a slight nonlinearity of the web deformation at the top chord are visible (Figure 14c). This is a sign of coupling between the transverse force *V* and torsion, rather than local plasticisation of the web, as seen in sample 4. The flanges enter the plastic range only in the final stage of failure (Figure 14e). In the initial stages, the curve is almost linear with an uneven distribution of the deformation across the width. The distribution of the deformation across the web height in the middle of the specimen span confirms the global torsion at the cross section (Figure 14b). At the measurement points located at the flanges, the negative and positive deformations systematically increase with increasing load. At the remaining points throughout the load range, the *ε* values are close to zero (Figure 14d). In both the lower and upper flanges, the curves are linear up to high load levels, as shown in Figure 14f. The distribution of *ε* is uneven across the width of the flanges, but without prior plasticisation. The development of deformations in girder sample 5 is consistent with the higher ultimate load capacity *F*_g_ and the observed form of failure. It is also consistent with the typical mechanical characteristics of an element in which shear stresses and transverse pressure are amplified by cross section rotation (LTB).

### 3.4. Discussion and Comparison with Previous Studies

In general, the static failure modes observed in the present study are consistent with previous experimental investigations on girders with corrugated webs [11,12,13,17,19]. The reference specimen and girder sample 4, which both failed by local web instability in the region of combined bending moment and shear, showed buckling patterns similar to those reported by Pasternak and Branka [11] and Dilger et al. [12] for girders with sinusoidal corrugated webs and by Sause et al. [13] and Kövesdi and Dunai [17] for trapezoidal corrugated webs. In all these studies, the first signs of instability appeared in the web panels adjacent to the high-moment/high-shear regions, followed by a progressive loss of shear stiffness and an increase in out-of-plane deformations of the web. The present tests confirm that this mechanism is also dominant for the investigated geometry and boundary conditions and that the corrugated web remains the critical component in terms of local buckling.

The global lateral–torsional buckling observed in girder sample 5 after long-term cyclic loading is also in line with the trends reported for laterally unrestrained corrugated-web girders [19,47]. Several authors have pointed out that the corrugated web contributes very little to the torsional rigidity of the cross section, so that the overall torsional stiffness is mainly provided by the flanges and transverse stiffeners [13,17,19]. As a consequence, corrugated-web girders are more susceptible to combined bending–torsion failure modes than comparable plate girders with flat webs. In the present study, this effect is clearly visible in girder sample 5, where residual torsional deformations accumulated during preliminary cyclic loading reduced the effective lateral and torsional stiffness, and the ultimate behaviour involved a pronounced interaction between local web deformation and global lateral–torsional buckling. This observation supports the conclusions of earlier works [19,47] and emphasises the need to explicitly consider lateral–torsional buckling in the design of sinusoidal corrugated-web girders, particularly when lateral restraints are limited or when significant cyclic pre-loading is expected.

The predominance of the load range Δ*F* over the number of cycles *N* as the main parameter governing fatigue damage in the present tests is consistent with the general fatigue design concepts in EN 1993-1-9 [26]. A similar tendency was reported in several experimental studies on corrugated-web girders, in which relatively high stress ranges led to early crack initiation and rapid damage accumulation even for moderate *N*, whereas specimens subjected to lower stress ranges survived a considerably larger number of cycles [13,17,19,22,24]. The present results confirm these trends for sinusoidal corrugated webs and for the specific welded details investigated. For the girders tested here, the change from a lower to a higher load range had a clearly more pronounced effect on stiffness degradation and residual ultimate capacity than the increase in the number of cycles at a given Δ*F*. This finding underlines the importance of accurately assessing the expected load ranges in service when evaluating the fatigue performance of corrugated-web girders.

In terms of classical high-cycle fatigue concepts, the two Stage 2 fatigue-to-failure tests provide two points on an *F*–*N* (or Δ*σ*–*N*, i.e., *S*–*N*) diagram for the investigated welded details. However, the present experimental matrix is far too limited to construct a full fatigue curve or to identify an endurance limit. In current design practice, for example, in EN 1993-1-9 [26], the reference fatigue strength of steel details is typically defined at a reference number of 2 × 10^6^ cycles, whereas the numbers of cycles applied in the present tests were lower, of the order of 10^5^–10^6^. The results presented here should therefore be interpreted as describing short- to medium-term fatigue behaviour under relatively high stress ranges, rather than as a complete characterisation of the long-life fatigue performance of the details.

Furthermore, the measured hot-spot stresses at the web–flange and web–stiffener welds confirm that nominal stress-based design approaches tend to be conservative for the investigated details, in agreement with observations reported in the literature for other welded joints [18,20,41,45,46]. In the present tests, the hot-spot stresses were significantly higher than the corresponding nominal stresses calculated on the basis of the gross cross section, which is consistent with the strong stress concentration caused by the geometric discontinuity at the weld toes. Nonetheless, when fatigue assessment is performed using nominal stress ranges and standard *S–N* curves as in EN 1993-1-9 [26], the predicted fatigue life tends to be on the safe side for the details considered. This suggests that, for practical design purposes, nominal stress-based methods can still be used for sinusoidal corrugated-web girders, provided that appropriate detail categories and partial safety factors are adopted.

Overall, the present results complement previous studies by providing full-scale experimental evidence on the interaction between local web instability, global lateral–torsional buckling and fatigue damage in girders with sinusoidal corrugated webs. In contrast to many earlier programmes focused solely on static behaviour or on cyclic tests without subsequent static failure tests, the combination of long-term cyclic loading and post-fatigue static tests used here makes it possible to directly assess the residual stiffness and residual ultimate capacity of the girders after different loading histories. This additional information is particularly relevant for the assessment of existing bridges and industrial structures subjected to repeated traffic or crane loads, where the structure may already have experienced a substantial part of its fatigue life. The findings presented in this paper therefore provide a useful experimental basis for calibrating numerical models and for developing more refined design and assessment procedures for sinusoidal corrugated-web girders.

## 4. Conclusions

This paper has presented an experimental study on full-scale steel girders with sinusoidal corrugated webs subjected to static and cyclic four-point bending. Based on the results obtained from five girders tested in different loading scenarios, the following main conclusions can be drawn:

1. Failure mechanisms. Two main failure mechanisms were observed: (I) local buckling of the sinusoidal web combined with yielding of the flanges, and (II) a combined failure involving local web buckling and global lateral–torsional buckling after prior cyclic loading. The second mechanism occurred only in the pre-fatigued specimen with significant residual torsional deformations.
2. Influence of fatigue loading parameters. For the tested girders and within the investigated ranges of stress ranges and numbers of cycles, the load range Δ*F* had a more pronounced influence on fatigue damage, stiffness degradation and residual ultimate capacity than the variation in the number of cycles *N*. Higher load ranges led to a stronger reduction in residual capacity even when the total number of cycles was relatively low. These observations are specific to the present tests and are not intended to replace the general *S*–*N*/*F*–*N* description used in fatigue design.
3. Residual stiffness and role of transverse stiffeners. Despite the accumulation of fatigue damage in the web and flanges, the global stiffness in the elastic range remained almost unchanged up to the late stages of loading, and the girders retained a significant portion of their original ultimate load after pre-fatigue. The use of shortened transverse stiffeners under the concentrated loads allowed the buckled web zone to propagate beyond the stiffener location, resulting in slightly conservative ultimate loads compared with an equivalent configuration with full-depth stiffeners.
4. Torsional effects and implications for design. The corrugated web contributed little to the torsional stiffness of the cross section, so torsional resistance was mainly provided by the flanges and transverse stiffeners. In most tests torsional effects had a limited influence on the bending capacity, but in the pre-fatigued specimen exhibiting lateral–torsional buckling, they interacted with local web buckling and reduced the ultimate load. The results highlight the need to consider lateral–torsional buckling and realistic load ranges in the design and assessment of sinusoidal corrugated-web girders. Further experimental and numerical studies, including a wider range of load ranges and numbers of cycles up to and beyond 2 × 10^6^ cycles, are recommended to characterise long-life fatigue behaviour and to support more refined design rules.

## Figures and Tables

**Table 1:** Geometry of the tested samples (unit: mm).

Length	Calculated Length	Height	Flange	Corrugated Web	Wavelength	Wave Amplitude	Wave Height	Developed Wavelength
*L*	*L*_0_	*h*	*t*_f_ × *b*_f_	*t*_w_ × *h*_w_	*q*	*a_f_*	*h*_s_	*S*
6510	6045	540	20 × 200	3 × 500	155	21.5	43	178

**Table 2:** Mechanical properties of S355 steel.

Location	Thickness	Young’s Modulus	Yield Strength	Tensile Strength
	[mm]	[GPa]	[MPa]	[MPa]
Corrugated web	3	207	370	506
Flange	20	209	379	514

**Table 3:** Parameters for plastic strengthening.

Location	Tensile Strain Hardening Exponent	Strength Coefficient
	[-]	[MPa]
Corrugated web	0.1589	779
Flange	0.1494	757

**Table 4:** Cyclic load parameters of the tested specimens.

Specimen	*F*_min_	*F*_max_	Δ*F*
	[kN]	[kN]	[kN]
2	10	260	250
3	10	130	120
		195	185
4	10	260	250
5	10	195	185

**Table 5:** Limit values for specimens 1, 4, and 5.

Specimens	Load History	*F*_g_	0.85*F*_g_	*δ*_max_	*δ*_0.85*F*g_	*F*_gsamples1_/*F*_gi_
		[kN]	[kN]	[mm]	[mm]	[%]
1	static only	323.62	275.08	91.82	6.87	100
4	223,200 *N* (Δ*F* = 250 kN) + static	175.03	148.78	65.47	2.54	54.09% (−45.91%)
5	1,250,000 *N* (Δ*F* = 185 kN) + static	184.40	156.74	79.76	3.37	56.98% (−43.02%)

## Data Availability

The original contributions presented in the study are included in the article, further inquiries can be directed to the corresponding author.

## Abbreviations

The following abbreviations are used in this manuscript:

*a*_f_I	Wave amplitude
*b*_f_	Flange width
*E*	Young’s modulus
*f*_u_	Tensile strength
*f*_y_	Yield strength
*n*	Tensile strain hardening exponent
*C*	Strength coefficient
*h*	Girder height
*h*_s_	Wave height
*h*_w_	Web height
*L*	Girder length
*L*_0_	Calculated girder length
*q*	Wavelength
*S*	Developed wavelength
*t*_f_	Flange thickness
*t*_w_	Web thickness
Δ*F*	Load amplitude
*N*	Number of cycles to failure
*σ*_nom_	Nominal stress
*σ*_hs_	Design stress
*M*	Bending moment
*V*	Shear force
